# The Bone Marrow Microenvironment in B-Cell Development and Malignancy

**DOI:** 10.3390/cancers14092089

**Published:** 2022-04-22

**Authors:** Anastasia M. Hughes, Vincent Kuek, Rishi S. Kotecha, Laurence C. Cheung

**Affiliations:** 1 Leukaemia Translational Research Laboratory, Telethon Kids Cancer Centre, Telethon Kids Institute, Perth, WA 6009, Australia; anastasia.hughes@telethonkids.org.au (A.M.H.); vincent.kuek@telethonkids.org.au (V.K.); rishi.kotecha@health.wa.gov.au (R.S.K.); 2 Curtin Medical School, Curtin University, Perth, WA 6102, Australia; 3 School of Biomedical Sciences, University of Western Australia, Perth, WA 6009, Australia; 4 School of Medicine, University of Western Australia, Perth, WA 6009, Australia; 5 Department of Clinical Haematology, Oncology, Blood and Marrow Transplantation, Perth Children’s Hospital, Perth, WA 6009, Australia; 6 Curtin Health Innovation Research Institute, Curtin University, Perth, WA 6102, Australia

**Keywords:** B-cell development, B-cell acute lymphoblastic leukemia (B-ALL), bone marrow microenvironment (BMM), B-cell niche, bone marrow (BM), leukemia

## Abstract

**Simple Summary:**

B cells are an essential component of the immune system and develop in the bone marrow microenvironment. B-cell development is tightly regulated by the stromal cells, fat cells and bone cells in this microenvironment. However, when B-cell malignancies arise, leukemic cells can alter normal microenvironment functioning to aid their growth, survival and resistance to cytotoxic therapies. This review summarizes the role of the bone marrow microenvironment in regulating healthy B-cell development and B-cell acute lymphoblastic leukemia (B-ALL). Understanding of how the microenvironment contributes to B-ALL pathogenesis and treatment failure will allow us to devise microenvironment-targeted therapies for B-ALL in the future.

**Abstract:**

B lymphopoiesis is characterized by progressive loss of multipotent potential in hematopoietic stem cells, followed by commitment to differentiate into B cells, which mediate the humoral response of the adaptive immune system. This process is tightly regulated by spatially distinct bone marrow niches where cells, including mesenchymal stem and progenitor cells, endothelial cells, osteoblasts, osteoclasts, and adipocytes, interact with B-cell progenitors to direct their proliferation and differentiation. Recently, the B-cell niche has been implicated in initiating and facilitating B-cell precursor acute lymphoblastic leukemia. Leukemic cells are also capable of remodeling the B-cell niche to promote their growth and survival and evade treatment. Here, we discuss the major cellular components of bone marrow niches for B lymphopoiesis and the role of the malignant B-cell niche in disease development, treatment resistance and relapse. Further understanding of the crosstalk between leukemic cells and bone marrow niche cells will enable development of additional therapeutic strategies that target the niches in order to hinder leukemia progression.

## 1. Introduction

Hematopoiesis is a process whereby multipotent and self-renewing hematopoietic stem cells (HSCs) progress to become mature lymphoid and myeloid blood cells in the bone marrow (BM) through differentiation in a hierarchically-organized and tightly-regulated manner. This process is sustained throughout life by a pool of quiescent and self-renewing HSCs, which act as a reservoir for actively proliferating and differentiating cells [1]. B lymphopoiesis is characterized by progressive loss of multipotent potential in HSCs, followed by commitment to differentiate and form B cells, a group of antibody-producing cells specialized in mediating the humoral response of the adaptive immune system. While the exact stepwise process of hematopoiesis remains under debate, in summary, B lymphopoiesis begins with differentiation of HSCs into multipotent progenitors (MPPs), which then give rise to lymphoid primed MPPs [2] and, subsequently, to common lymphoid progenitors (CLPs) [3,4]. The commitment of CLPs towards B-cell lineage is designated by expression of Ly6D, a surface marker that identifies the first stage of B-cell lineage-specific development [5]. This is then followed by sequential differentiation of CLPs into pre-pro-B cells, pro-B cells, large pre-B cells, small pre-B cells and immature B cells in the BM. These populations are characterized by their expression of stage-specific surface receptors, adhesion molecules and sequential recombination events, which lead to formation of the B-cell receptor [6,7,8]. 

The BM microenvironment (BMM) plays a vital role in hematopoiesis and B-cell development. It is a region enriched with arterioles, sinusoidal blood vessels, sympathetic nerve fibers, and a myriad of BMM-derived regulatory signals, which control and define the fate of the hematopoietic and mesenchymal cell lineages that reside within [1]. Importantly, BM niches have been implicated in driving hematological malignancies, with essential roles in disease development, progression and treatment resistance [9]. Thus, development of therapeutic strategies that disrupt malignant cell-BM niche interactions is of significant interest to scientists and clinicians. In this review, we discuss the cellular constituents of these BM niches and their involvement in B lymphopoiesis. We also outline the role of these niche cells in B-cell acute lymphoblastic leukemia (B-ALL) and the regulatory mechanisms involved.

## 2. The Bone Marrow Microenvironment in B-Cell Development

**Figure 1:**
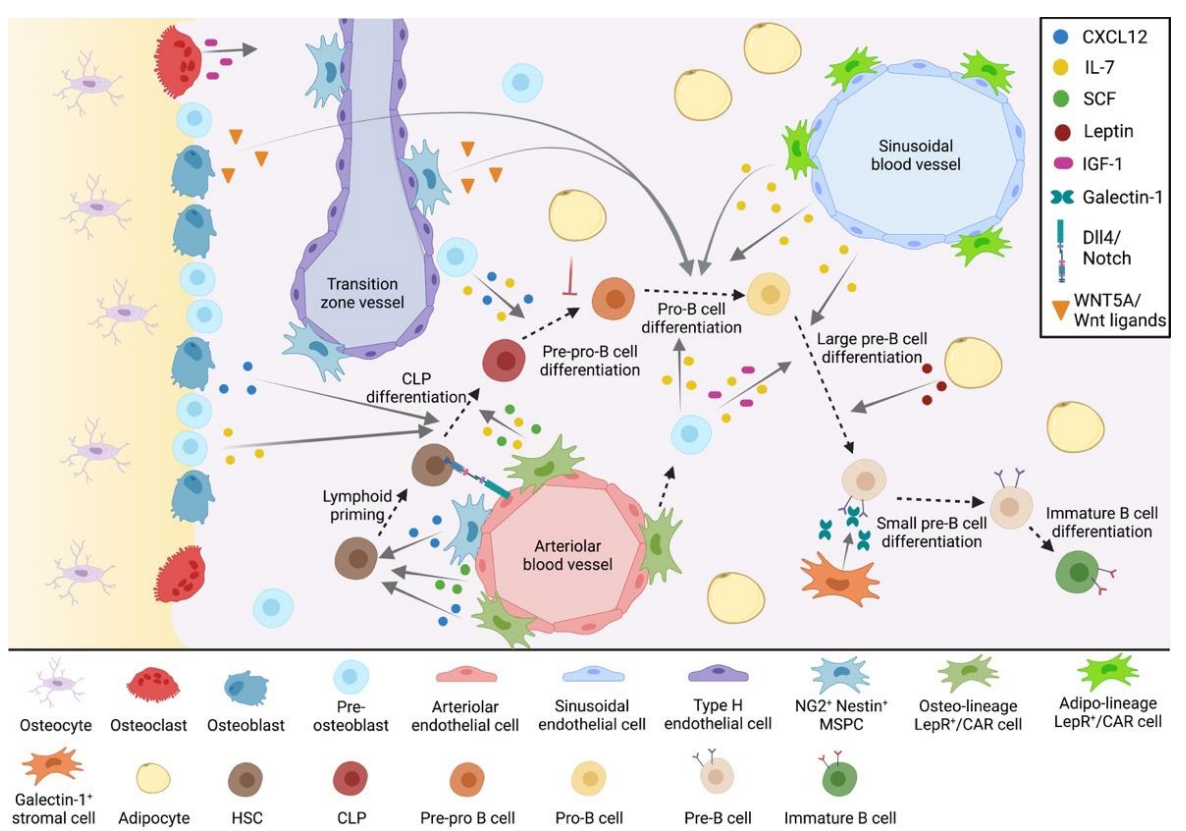
**The progression of B-cell development within the bone marrow microenvironment.** Cells within the bone marrow microenvironment drive B lymphopoiesis by providing lineage instructive cues to B-cell progenitor populations. These cues are integrated at distinct developmental stages as B-cell progenitors move between bone marrow niches. Abbreviations: C-X-C motif chemokine ligand 12 (CXCL12); interleukin-7 (IL-7); stem cell factor (SCF); insulin-like growth factor-1 (IGF-1); delta-like 4 (Dll4); Wnt Family Member 5A (Wnt5A); mesenchymal stem and progenitor cell (MSPC); leptin receptor (LepR); CXCL12 abundant reticular (CAR); hematopoietic stem cell (HSC); common lymphoid progenitor (CLP).

B-cell development is regulated by spatially distinct BM niches. The current HSC niche model consists of two distinct niches; endosteal and central, which are discernible by their location and cellular composition. The endosteal niche is enriched with osteoblasts and osteoclasts, and has recently been identified to contain transition zone blood vessels composed of endothelial cells and surrounded by perivascular mesenchymal stem and progenitor cells (MSPCs) [10]. In contrast, the central niche contains perisinusoidal and periarteriolar blood vessels made up of endothelial cells and surrounded by perivascular MSPCs [10]. The endosteal and central niche both have roles in B-cell development. (Figure 1)

### 2.1. The Endosteal B-Cell Niche

Using rat and mouse models, studies have provided anatomical evidence of a preferential localization of early B-cell precursors in the subendosteal region of the BM [11,12,13,14]. This implies existence of an endosteal niche, where bone remodeling cells such as bone-forming osteoblasts and bone-resorbing osteoclasts are localized, in addition to recently discovered transition zone vessels. Interestingly, other studies have contradicted these findings, noting that greater than 80% of CLPs were positioned >30 µm away from the endosteum [15]. While the importance of the endosteal niche in B lymphopoiesis remains under debate, current findings suggest that several cell types in the endosteal niche have a specific role in B-cell development (Figure 1).

#### 2.1.1. Osteoblasts 

In recent years, the role of osteoblasts in B lymphopoiesis has been intensively investigated. For instance, a study found that osteoblasts were able to induce lineage commitment of primitive HSCs into IgM^+^ immature B cells during in vitro co-culture, providing evidence of the supportive role of osteoblasts in B lymphopoiesis [16]. Furthermore, in vivo ablation of both Col1α1-2.3kb (*Col*2.3)-targeted mature osteoblasts [16,17] and osterix (*Osx*)-targeted pre-osteoblasts [18,19] was found to impede B lymphopoiesis. Reduced B lymphopoiesis was observed along with minimal perturbation to hematopoietic stem and progenitor cell (HSPC) numbers, indicating a distinct regulatory influence on B-cell progenitor subsets rather than HSPCs [16,18,19]. To gain further insight into the contribution of osteolineage cells to B lymphopoiesis, various studies have conditionally attenuated expression of essential B-cell niche factors, including CXC chemokine ligand 12 (CXCL12), interleukin-7 (IL-7), insulin-like growth factor-1 (IGF-1) and Wnt Family Member 5A (WNT5A), in osteolineage cells. 

CXCL12 has been identified as a major factor for B-cell development and is expressed in osteolineage populations [11,20,21]. A profound impact on B lymphopoiesis was observed with conditional deletion of *Cxcl12* in pre-osteoblasts, mediated by the *Osx-Cre* transgene, resulting in reduction of B lineage progenitors from the pre-pro-B differentiation stage onwards [22]. However, this study noted that in *Osx-Cre* targeted mice, *Cxcl12* expression was also reduced in the CXCL12 abundant reticular (CAR) cell population; thus, the observed impact on B-lymphopoiesis could not be attributed to osteolineage cells alone. Another study found that attenuating expression of *Cxcl12* in mature osteoblasts using *Col2.3-Cre* induced modest but significant reductions in early lymphoid committed progenitor populations, but not in downstream B-cell progenitors [11]. In contrast, deletion of *Cxcl12* in mineralizing mature osteoblasts, mediated by the osteocalcin (*Ocn)-Cre* transgene, had no effect on the frequency of lymphoid progenitors [22]. These confounding findings may be a result of the different transgenic models used to target mature osteoblasts and, hence, recombination occurring in different populations of osteolineage cells. Overall, while current evidence suggests that CXCL12 produced by early osteoprogenitors and, to a lesser extent, COL2.3^+^ mature osteoblasts may be important for B lymphopoiesis, transgenic mouse models with the ability to target more specific osteolineage populations will be essential for confirmation of these findings.

IL-7 is another important B-cell growth factor that is indispensable for differentiation and maturation of B lymphocytes [23]. While mature osteoblasts do not appear to provide an essential source of IL-7 to the B-cell niche [15], reduction of *Il7* expression in the *Osx-cre*-targeted pre-osteoblastic population significantly impaired B lymphopoiesis at multiple developmental stages in several studies [18,24,25,26,27]. Another cytokine, IGF-1, was identified as a potential B-cell niche factor when it was observed to support the development of pro-B cells from HSPCs in vitro [28]. The endosteal niche has been identified as a potential source of this cytokine, with pre-osteoblasts [19] and COL2.3^+^ osteoblasts [29] both found to express IGF-1. Deletion of IGF-1 in OSX^+^ cells was found to inhibit the pro-B to pre-B transition, which suggests that pre-osteoblast-derived IGF-1 is indispensable for B lymphopoiesis [19]. 

Wnt signaling has also been implicated in regulating hematopoiesis and B lymphopoiesis in the BM [30,31]. WNT5A is known to be expressed in the endosteal niche by COL2.3^+^ osteoblasts [29]. Furthermore, the osteoblast-specific impairment of Wnt protein secretion, mediated by *Wntless* deficiency in *Col1-Cre* transgenic mice, resulted in impaired pro-B, pre-B and immature B cell numbers within the BM, and reduced IL-7 levels [31]. Although Wnt signaling is an important mediator of B lymphopoiesis in the endosteal niche, it remains unclear whether Wnt proteins regulate B lymphopoiesis directly or indirectly via modulation of other B-cell niche factors, and further research is required to address this question. For example, Wnt is known to be a critical regulator of osteoblast formation [32]; therefore, impaired Wnt protein secretion may disrupt BM osteoblasts, thus altering the endosteal B-cell niche and exerting an indirect impact on B lymphopoiesis. 

Overall, whilst osteoprogenitors and osteoblasts are vitally important in B-cell development through their production of lymphoid niche factors, their roles in this process appear to be highly dependent on the stage of osteogenesis.

#### 2.1.2. Osteocytes

Osteocytes are terminally-differentiated osteolineage cells that reside within the lacunae of mineralized bone matrix. They comprise between 90–95% of all bone cells and contribute to maintenance of bone homeostasis via interactions with osteoclasts and osteoblasts. It has been reported that osteocytes are capable of modulating osteoclast differentiation indirectly by influencing the expression of receptor activator of NF-kB ligand (*Rankl*) in osteoblasts [33]. Furthermore, osteocytes can directly inhibit osteolineage differentiation of mesenchymal progenitors and indirectly promote bone resorption via secretion of sclerostin [34]. Intriguingly, mice harboring a global deletion of sclerostin showed increased osteoblast activity [35] and an osteopetrotic phenotype [36], which was accompanied by decreased expression of *Cxcl12* in BM stromal cells and elevated apoptosis of B-cell progenitors in the BM [36]. It is worth noting that the same study also demonstrated that sclerostin is primarily expressed in osteocytes and not in hematopoietic lineage cells, implicating a non-cell autonomous impairment of B-cell differentiation by osteocytes. Similarly, conditional ablation of the osteocyte population using transgenic mice engineered to express the diphtheria toxin receptor under the dentin matrix protein 1 (*Dmp1*) promoter indirectly induced lymphopenia by depleting lymphoid-supportive stroma in the BM [37]. Therefore, it is plausible to conclude that osteocytes play a critical role in the regulation of B-cell niche populations. Whether osteocytes can directly stimulate B-cell lymphopoiesis through direct intercellular crosstalk remains to be investigated.

#### 2.1.3. Osteoclasts

Osteoclasts are large multinucleated cells that are responsible for breaking down bone tissue during bone remodeling and repair. The differentiation and maturation of osteoclasts is mediated largely by binding of RANKL to the receptor activator of NF-kB (RANK), which is expressed on osteoclast precursors [38]. It has been reported that depletion of osteoclasts within the BM via knock-out of RANKL [39] or its receptor RANK [40] can lead to severe osteopetrosis, which is also accompanied by defects in B lymphopoiesis. In other studies where osteoclast-mediated bone resorption was inhibited either through genetic or pharmacological approaches, impairment to B lymphopoiesis was also detected [41,42]. This effect appeared to be caused by CXCL12 and IL-7 depletion in the B-cell niche, possibly due to a reciprocal decrease in osteoblast differentiation as a result of abrogated osteoclastic activity [42]. While it remains unclear whether osteoclasts can directly affect B-cell development, several important matrix-derived B-cell niche factors such as IGF-1 [43] are known to be activated and released into the BMM during bone resorption. The role that this process plays in B lymphopoiesis is worthy of further investigation. Overall, current evidence of a direct crosstalk mechanism between osteoclasts and B cells is lacking; however, the indirect impact of osteoclastic activity on B lymphopoiesis through regulation of other B-cell niche populations remains plausible. 

### 2.2. The Central B-Cell Niche 

The central BM niche comprises 90% of total BM volume and contains an extensive vascular network, which is composed of a central artery and vein, as well as arterioles that connect to a network of sinusoidal vessels via transition zone vessels at the endosteum [10]. These blood vessels are made up of endothelial cells, which are in contact with perivascular MSPCs and sympathetic nerve fibers. Perivascular MSPCs are overlapping cell populations that include CAR cells [21], leptin receptor (LepR^+^) MSPCs [44], nestin^+^ MSPCs [45], neural glial antigen 2 (NG2^+^) MSPCs [46] and PDGFR-α^+^ Sca1^+^ (PαS) MSCs [47]. Additionally, galectin-1^+^ stromal cells [48] and adipocytes [49] are considered essential components of this system. Collectively, these cells make up the central hematopoietic niche (Figure 1).

#### 2.2.1. Perivascular MSPCs 

Mesenchymal stem cells (MSCs) are known to possess tri-lineage differentiation potential and are capable of giving rise to osteoblastic, adipocytic and chondrogenic cell lineages in the BM. As these stem cells undergo differentiation, they transition into early mesenchymal progenitor populations, which are capable of committing to a single lineage, dictated by lineage-specific internal/external stimuli. The supportive role of these MSPCs in hematopoiesis was illustrated over a decade ago by mouse studies, wherein sub-cutaneous or sub-renal injection of MSPCs generated an ectopic BMM that supported host hematopoiesis [50,51]. Furthermore, analysis of BM sections by microscopy has identified that B-cell progenitors, at multiple developmental stages, are in contact with CAR cells [52], LepR^+^ MSPCs [53] and IL-7/CXCL12 expressing MSPCs [54], suggesting that these stromal cells form an important niche that is supportive of B-cell development. 

Identification of perivascular MSPC populations commonly relies on their close proximity to the BM vasculature and expression of factors/markers such as LepR, nestin, CXCL12, IL-7, stem cell factor (SCF), NG2 and paired-related homeobox 1 (PRX1) [8]. Transgenic reporters or conditional knockout mouse models that target promoter sequences of MSPC-related genes enable these populations to be identified, ablated or their production of certain niche factors silenced, thus allowing functional assessment of their roles in B lymphopoiesis. A summary of these markers and cre-recombinase mouse strains has been documented [8]. The conditional ablation of the CAR cell population through diphtheria toxin receptor knock-in at the *Cxcl12* locus resulted in significantly reduced CXCL12 and SCF levels in the BM, reduced numbers of CLPs and pro-B cells, and increased apoptosis and quiescence in the pro-B cell population [55]. These results demonstrated an essential role for the CAR cell population in B lymphopoiesis. 

In the BMM, cytokines produced by perivascular MSPCs are indispensable for normal B-cell development. For instance, deletion of *Cxcl12* in *Prx1-Cre* expressing stromal cells, a cell population which includes LepR^+^ MSPCs [56], CAR cells and osteoblasts [22], significantly impaired B lymphopoiesis, as shown by reductions in B-cell progenitor populations in the BM [11,22]. Furthermore, conditional knock-out of *Cxcl12* in a subset of the LepR/CAR population using an *Il7-Cre* transgenic mouse model induced reductions in HSCs and MPPs within the BM [15]. Similar findings were observed in a *LepR-Cre* mouse model, with conditional deletion of *Cxcl12* in LepR^+^ perivascular MSPCs impairing HSC retention in the BM [11]. In contrast, *Nestin-Cre* mediated *Cxcl12* deletion did not induce any hematopoietic defects, indicating that the nestin^+^ MSPC population does not provide an essential source of this cytokine for hematopoiesis [11]. However, discrepancies between the expression of nestin transgenes have been observed, with *Nestin-GFP* appearing to be expressed in a different subpopulation of MSPCs compared to other nestin transgenes [44]. Therefore, the effect of *Nestin-Cre*-directed *Cxcl12* deletion may not be representative of the entire nestin^+^ MSPC population. Interestingly, LepR^+^ MSPCs have been reported to be a major source of IL-7 in the BMM [15]. Deletion of *Il7* in a *LepR-Cre* transgenic model has been shown to reduce the number of Ly6D^+^ CLPs, leading to markedly-reduced lymphoid progenitors in the BM and subsequent development of B lymphopenia [15]. In addition, *Prx1-Cre* targeted deletion of *Il7* was also shown to affect B-cell development at the pro-B stage [15]. Wnt ligand secretion by MSPCs has also been identified as an important factor in the perivascular B-cell niche. *Wntless* ablation in a *Nestin-Cre* mouse model identified that Wnt ligand production by nestin^+^ MSPCs is important for B lymphopoiesis from the pro-B stage of development onwards [31]. Another niche factor, connective tissue growth factor (CTGF), was shown to be important for hematopoiesis, with *Ctgf* knock-out mice exhibiting deficiencies in B lymphopoiesis [57]. Further investigation in vitro found that in the presence of IL-7, CTGF facilitated the pro-B to pre-B transition and proliferation of both progenitor populations. CTGF is expressed by BM stromal cells including MSCs and CAR cells; thus, is likely to play an important role in the central B-cell niche [57]. However, further work delineating stromal subtype-specific effects of CTGF on B lymphopoiesis is needed. Finally, *LepR-Cre* specific deletion of *Scf* in LepR^+^ cells reduced HSC, MPP and CLP numbers, while downstream progenitors remained unaffected [58]. Similarly, *Cre*-induced knockout of *Scf* in IL-7^+^ cells led to a reduction in HSC and MPP populations; however, downstream progenitor numbers were not assessed [15]. Overall, these results suggest that LepR^+^ MSPCs, PRX1^+^ MSPCs and CAR cells are important sources of CXCL12 and IL-7 for B lymphopoiesis, while their production of SCF appears to be critical at earlier stages of B lymphopoiesis. 

It is important to note that earlier studies utilized markers for perivascular MSPC populations that have since been found to encompass a heterogenous stromal cell population. Through single cell RNA sequencing technologies and advances in microscopy, these populations have been found to vary in their gene expression, cytokine expression, lineage priming and location within the BMM. As such, an important distinction has been drawn between perisinusoidal and periarteriolar niches. LepR^+^, Nestin-GFP^dim^ MSPCs and CAR cells appear to be overlapping perivascular stromal populations [56], but can be further classified into perivascular subpopulations depending on their BM anatomical location around sinusoids or arterioles, which exhibit adipogenic or osteogenic lineage priming, respectively [12,29,59,60]. In addition, the Nestin-GFP^bright^, NG2^+^ MSPC population, which localizes around arterioles [46], is distinct from LepR^+^ and CAR populations and is thought to sit at the top of the MSPC differentiation hierarchy [60]. This NG2^+^ MSPC population has also been identified in the proximity of type H/transition zone vessels [61]. Interestingly, recent investigations have found that periarteriolar MSPCs may provide a niche for a quiescent population of lymphoid-biased HSCs, with NG2^+^ cell depletion reducing this HSC population by half [62]. Finally, osteolectin has recently been identified as a surface marker specific for arteriolar LepR^+^ MSPCs [12]. This population forms an important lymphoid niche, with 35% of CLPs localized within 5 μm of osteolectin^+^ LepR^+^ cells. SCF production by this niche population is essential, as its deletion led to a drastically-reduced frequency of CLPs, pre-pro-B cells and pre-B cells in the BM [12]. Taken together, current data suggests that periarteriolar NG2^+^ nestin-GFP^bright^ MSPCs support lymphoid-biased HSCs, while periarteriolar LepR^+^ osteolectin^+^ MSPCs contribute to a SCF-rich niche for the earliest lymphoid progenitors.

In contrast to periarteriolar MSPCs, perisinusoidal MSPCs have not been well studied, and their importance in B lymphopoiesis has only recently been appreciated. In the BM, adipocytic-primed LepR^+^ cells associated with sinusoidal capillaries (as opposed to periarteriolar, osteogenic-primed LepR^+^ cells) [12] are highly enriched for pro-hematopoietic factors such as CXCL12, SCF and IL-7 [29]. Pro-B cells have been shown to localize around IL-7^high^ perisinusoidal LepR^+^ cells, away from the endosteum [53]. This indicates that the perisinusoidal niche may be required for committed B-cell progenitors. Therefore, it is plausible that progression of B-cell development requires trafficking of early B-cell progenitors to the perisinusoidal niche. Future studies delineating the potential differential contributions of arteriolar/sinusoidal MSPCs to B lymphopoiesis within the central niche will be worthwhile. 

#### 2.2.2. Galectin-1^+^ Stromal Cells 

Research has identified stromal cells that express galectin-1, a pre-B-cell receptor ligand essential for proliferation and differentiation of large pre-B cells [63]. Interestingly, galectin-1^+^ has also been identified in a stromal cell subset lacking IL-7 expression [48]. This population was distributed evenly throughout the BM with no apparent association with vascular structures and could therefore represent a distinct B-cell niche for large pre-B cells as they transition to small pre-B cells. 

#### 2.2.3. Endothelial Cells

Blood vessels are composed of endothelial cells, which form a critical cellular component of the BMM and regulate the exchange of cells, nutrients, soluble factors, oxygen and waste between the BMM and peripheral blood. BM vasculature can be classified based on surface marker expression levels of endomucin and CD31 on endothelial cells. Research has identified type H transition zone vessels (endomucin^high^, CD31^high^), which are fed directly by arterioles in the metaphysis and endosteum, and type L (endomucin^lo^, CD31^lo^) sinusoidal vessels predominantly associated with central BM [64]. Similarly, single cell RNA sequencing has identified two BM VE-Cadherin^+^ endothelial cell clusters that differentially profile based on their sinusoidal or arterial gene signatures [29]. Sinusoidal and arteriolar endothelial cells not only differ in properties such as permeability and surface marker expression [65], but also in their supportive abilities for hematopoiesis. 

Endothelial cells are known to contribute hematopoietic cytokines such as CXCL12 [11,20,22,66], IL-7 [15], SCF [44] and CTGF [57] to perivascular BM regions. For instance, several studies have shown that deletion of *Cxcl12* [11,22] and *Scf* [44,58] from endothelial populations using the *Tie2-Cre* transgene could impair HSPC maintenance to a certain extent without significantly affecting B lymphopoiesis. In contrast, *Il7* deletion driven by the *Tie2-Cre* transgene reduced pro-B and pre-B cell populations, suggesting that endothelial cell-derived IL-7 is important for B lymphopoiesis [15]. However, the use of *Tie2* as an endothelial-specific promoter in driving gene deletions cannot conclusively distinguish contributions by different subtypes of endothelial cells. To overcome this, a recent study differentiated arteriolar endothelial cells from sinusoidal endothelial cells based on their selective expression of *Bmx* and *Epor*, respectively [67]. The same study also revealed that *Cxcl12* and *Scf* expression was significantly higher in arteriolar endothelial cells compared to sinusoidal endothelial cells. While arteriolar endothelial cell-derived SCF was required for HSC maintenance and regeneration, *Scf* deletion in either endothelial subtype had no effect on CLP numbers, with impact on more committed B-cell progenitors not assessed in this study [67]. Intriguingly, another subset of BM arteriolar endothelial cells that uniquely express von Willebrand factor were recently characterized and found to express the highest levels of *Scf* compared to other endothelial subsets [59]. Whether this new subset of arteriolar endothelial cells is important in regulating B-cell development remains to be investigated.

Finally, endothelial cells are known to regulate B lymphopoiesis through the Notch signaling pathway. The Notch signaling pathway is highly conserved and functions to regulate cell fate during tissue development through interactions between the transmembrane Notch receptor and Notch ligands Delta and Jagged [68]. In the BM, VE-Cadherin^+^ endothelial cells express the highest levels of the delta-like Notch ligands *Dll4* and *Dll1*, in comparison with COL2.3^+^ osteoblasts and LepR^+^ MSPCs [29]. Deletion of *Dll4* in endothelial cells induced a myeloid bias in HSPCs, reducing CLP numbers and, ultimately, B220^+^ B cells and CD3^+^ T cells [29]. This provides evidence for a role of endothelial cell-derived Dll4 in directing lymphoid differentiation of HSPCs. 

Overall, current data suggests that endothelial cells play a vital role in supporting B lymphopoiesis, either directly through IL-7 secretion or indirectly via the production of CXCL12/SCF to regulate the earliest hematopoietic progenitors and induce lymphoid lineage commitment of HSPCs through Dll4.

#### 2.2.4. Adipocytes

BM adipocytes differentiate from MSPCs in perivascular BM niches [56] and comprise 45% of total BM volume in regions where hematopoiesis and bone remodeling are active [69]. Research into marrow adipose tissue has advanced significantly in recent years and this tissue is now considered to be a complex endocrine organ that actively participates in hematopoiesis instead of simply “filling” the marrow [70]. Interestingly, contrasting evidence exists for the influence of adipocytes on B lymphopoiesis, which is likely to result from the vast array of cytokines, termed adipokines, secreted by this population. Over the years, evidence has pointed to the role of adipocytes as negative regulators of hematopoiesis, with HSC frequency found to be lower in bones with high marrow adipose tissue content, such as tail vertebrae in mice [71]. Similarly, mice receiving a high-fat diet leading to increased marrow adiposity exhibited significantly-reduced B-cell frequency and *Il7* expression in the BM [72]. In vitro studies have also highlighted the negative impact of adipocytes on B lymphopoiesis, with adipocyte-derived factors capable of blocking B-cell development at the CLP to pre-pro-B transition [73]. In addition, adipocytes have been found to drive differentiation of myeloid-derived suppressor cells in vitro, with pro-inflammatory factors secreted from this myeloid population (such as interleukin-1 (IL-1)) capable of suppressing B lymphopoiesis [74,75]. This phenomenon is also thought to contribute to the reduction of B lymphopoiesis in aging, where a concomitant increase in BM adipose tissue is observed [75]. Conversely, evidence from other studies suggests that certain adipokines are beneficial to B lymphopoiesis. In particular, leptin has been found to support B-cell development [76]. In a study of mice that lack functional leptin, there was a 50% reduction in the lymphoid compartment of the BM and a block in the differentiation of pro-B cells into more mature progenitors [77]. In these mice, normal B-cell development could be partially restored by leptin injections, indicating the essential role of this adipokine in B lymphopoiesis [77]. Therefore, current literature suggests that adipocytes have a multifaceted role in B lymphopoiesis. Furthermore, it is plausible to postulate that surrounding cells in the BMM could play a role in regulating adipokine secretion by adipocytes, and thus, further research is needed to dissect this possibility. 

## 3. The Bone Marrow Microenvironment in B-Cell Malignancy 

B-ALL arises from the uncontrolled, clonal proliferation of a B-cell progenitor population in the BM. Several well-characterized chromosomal alterations, such as *KMT2A* gene rearrangements or *BCR-ABL1* translocations, are responsible for driving clonal proliferation in leukemia initiating cells [78]. In addition, alterations in the transcriptome of leukemic cells are often observed in genes encoding transcription factors (e.g., *PAX5*, *IKZF1 (IKAROS)*, *EBF1*), surface receptors (e.g., *IL7R* and *FLT3*) and signal transduction proteins such as the Janus kinase (*JAK*) family that are known to regulate B lymphopoiesis [79,80,81]. While these intrinsic genetic aberrations can significantly impact a patient’s prognosis and treatment outcomes, accumulating evidence also points to the role of extrinsic concomitant alterations occurring in cellular constituents of the BMM that are capable of driving B leukemogenesis. 

Akin to normal hematopoiesis, BM niches can significantly influence the survival of leukemic cells [10]. Over the years, the development of genetically-engineered mouse models has enabled manipulation of cell populations within BM niches, allowing researchers to understand the role of the BMM in initiating hematological disease. In particular, such alterations have been found capable of facilitating malignant transformation of hematopoietic progenitors in the BM. Conversely, alterations to normal cells within BM niches can arise as a consequence of leukemic disease in the BM. These changes are believed to hinder hematopoiesis while also facilitating leukemia progression and treatment evasion. Thus, it is believed that cell populations in the BMM likely influence multiple stages of leukemia, both prior to and during overt disease [10,82,83]. While substantial progress has been made in understanding the influence of the BMM in myeloid malignancies [84], the BMM of B-lineage malignancies is less defined. 

### 3.1. The Endosteal Niche in B-ALL

**Figure 2:**
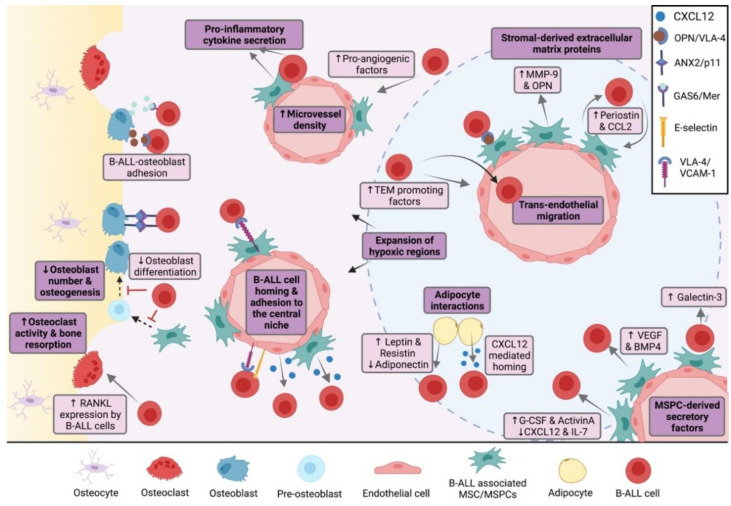
**Alterations induced in the bone marrow microenvironment by B-cell acute lymphoblastic leukemia (B-ALL).** B-ALL-associated microenvironment populations support the growth and survival of B-ALL cells and also provide chemoprotection from current frontline therapies. Pro-angiogenic factors include basic fibroblast growth factor, hepatocyte growth factor and tumor necrosis factor-α(TNF-α). Pro-inflammatory cytokines include TNF-α, interleukin-6, interleukin-8, interleukin-10, interleukin-12, interferon-γ and CC chemokine ligand 2 (CCL2). Trans-endothelial migration (TEM) promoting factors include cortactin, mDia1 and vascular endothelial growth factor (VEGF). Abbreviations: receptor activator of nuclear factor kappa-B ligand (RANKL); C-X-C motif chemokine ligand 12 (CXCL12); matrix metalloproteinase-9 (MMP-9); osteopontin (OPN); granulocyte colony-stimulating factor (G-CSF); interleukin-7 (IL-7); bone morphogenic protein (BMP4); very late antigen-4 (VLA-4); annexin II (ANX2); growth arrest-specific 6 (GAS6); vascular cell adhesion molecule-1 (VCAM-1); mesenchymal stem cell (MSC); mesenchymal stem and progenitor cell (MSPC).

In B-ALL, the BMM is often characterized by extensive remodeling prior to the initiation of treatment [85,86], which is known to be osteotoxic [87,88]. In a recent study, both osteoblasts and osteoclasts were found to be significantly reduced in BM trephines of children with B-ALL at diagnosis [89]. This study indicates that alterations to these bone cell populations, which are directly driven by the intercellular crosstalk between bone cells and B-ALL cells, could underlie the observed skeletal abnormalities. As such, leukemia-induced bone defects are increasingly being recognized, not only for their negative impact on a patient’s quality of life, but also for their importance in disease progression (Figure 2).

#### 3.1.1. Osteoblasts

While the role of osteoblasts in myeloid malignancies have been extensively characterized, their role in the B-ALL niche has only recently been appreciated [90]. The establishment of an immunocompetent syngeneic BCR-ABL1^+^ B-ALL mouse model that replicates bone loss in patients provides a useful tool for comprehensive investigation of the B-ALL microenvironment [91]. During leukemia development, reduction in the osteoblastic population and decreased serum levels of osteocalcin were observed [91]. Interestingly, direct impairment of osteogenesis by leukemic cells has been demonstrated by an in vitro study, which showed that B-ALL could inhibit the osteogenic differentiation of MSCs [92]. Furthermore, it is also possible that B-ALL could affect the apoptotic and/or cell cycle pathways of osteoprogenitors and osteoblasts. Thus, these potential mechanisms of B-ALL-mediated bone loss present a significant area of interest for future exploration using preclinical mouse models. 

Interestingly, alterations to the composition of the endosteal niche may also benefit leukemic cell survival and progression of disease. For instance, increased dormancy has been observed in B-ALL cells residing in the endosteal/osteoblastic niche of xenograft mouse models [93]. This was also confirmed using a microfluidics-based microphysiological culture system which recreated the B-ALL microenvironment in vitro [94]. A key mechanism facilitating B-ALL-endosteal niche interactions is via secretory factors. Osteoblasts are known to secrete osteopontin (OPN), an extracellular matrix (ECM) protein and endosteal adhesion molecule. OPN can bind to B-ALL cells through interaction with very late antigen-4 (VLA-4), resulting in their binding and anchoring to the endosteal niche [93]. This process leads to further upregulation of B-ALL and osteoblast-derived OPN, thus reinforcing endosteal adhesion and promoting leukemic cell dormancy [93]. The same study also showed that an OPN neutralizing antibody could block B-ALL-osteoblast interactions, resulting in an increase in proliferating blasts and rapid disease onset in vivo [93]. These results support the notion that the endosteal niche plays a role in modulating leukemogenesis, likely via hindering the proliferation and spread of B-ALL cells through induction of dormancy. 

In B-ALL, incomplete clearance of dormant leukemic cells by chemotherapy often leads to the persistence of minimal residual disease (MRD), which can subsequently lead to chemoresistance and relapse. Accumulating evidence points to the role of the endosteal niche in supporting B-ALL chemoresistance. Recent in vitro modeling showed that B-ALL cells localized near osteoblasts of the endosteal niche were more resistant to the cytotoxicities of prednisone, vincristine and nilotinib [94]. A key mechanism of leukemic cell resistance is through cell–cell adhesion contact between osteoblasts and B-ALL cells. For instance, osteoblasts express annexin II (ANX2), which forms a heterotetramer complex with p11, a surface protein that is highly upregulated in B-ALL cells of patients with relapsed disease [95]. Disruption of the ANX2/p11 complex with inhibitors in vitro abrogated osteoblast-mediated adhesion, thus sensitizing B-ALL to chemotherapy [95]. Interestingly, a subtype-specific mechanism of osteoblast-mediated chemoresistance via the growth arrest-specific 6 (GAS6)/Mer interaction has also been delineated in B-ALL expressing the *E2A-PBX1* translocation. GAS6 is an osteoblast-secreted ligand for receptor tyrosine kinase Mer, which is highly expressed in E2A-PBX1^+^ B-ALL cells [96]. Coculture of osteoblasts with a E2A-PBX1^+^ B-ALL cell line induced upregulation of GAS6 secretion by osteoblasts. The addition of GAS6 to cell culture media induced B-ALL cell chemotaxis via GAS6/Mer binding, which resulted in B-ALL quiescence and expression of anti-apoptotic factors, leading to chemoresistance [96]. Furthermore, another study has characterized a subpopulation of B-ALL cells that can migrate under the adherent osteoblastic cell layer and exhibit a dormant, chemotherapy-resistant phenotype [97]. This dormancy was found to be induced by downregulation of miR-221 and miR-222 in B-ALL cells, resulting in increased translation of their target gene p27, a cyclin dependent kinase inhibitor that blocks cell cycle progression at the G0/G1 transition [98]. Furthermore, the same group implicated the role of BCL-6, a cytokine signaling regulator, in B-ALL cell–osteoblast interactions. They noted a decrease in BCL-6 abundance in B-ALL cells co-cultured with osteoblasts, which consequently reduced B-ALL cell proliferation [99]. Forced expression of BCL-6 in B-ALL cells sensitized leukemic cells to chemotherapy, suggesting that disrupting B-ALL-osteoblast interaction by targeting BCL-6 could represent a viable treatment strategy [99]. 

#### 3.1.2. Osteoclasts

In an immunocompetent syngeneic mouse model of BCR-ABL1^+^ B-ALL, increased osteoclast activity was observed in the BM, coupled with increased bone resorption and progressive bone loss over disease development [91]. Notably, RANKL in B-ALL cells was 50–100-fold higher compared to non-malignant B cells and was shown to induce differentiation of osteoclasts in vitro. This indicates that the RANKL signaling pathway is a key mechanism through which B-ALL cells promote osteoclast activity and bone resorption in vivo. This finding was also confirmed by others, who showed that *RANKL* was detected in samples from patients with primary B-ALL and played a critical role in RANKL-dependent bone destruction in patient-derived xenograft (PDX) models of B-ALL [100]. Pharmacological inhibition of osteoclastic bone resorption with zoledronic acid [91], a bisphosphonate used for the treatment of osteoporosis, or with recombinant osteoprotegerin-Fc [100], an antagonist of RANKL, successfully prevented B-ALL-mediated bone loss in mouse models of B-ALL. Taken together, these results indicate that osteoclasts and the RANK-RANKL signaling axis are novel, promising targets for the treatment of B-ALL. 

#### 3.1.3. Endosteal Transition Zone Vessels

Leukemic cells are known to evade therapy by seeking “refuge” in the protective BM perivascular niche, where the vasculature often plays a role in facilitating crosstalk with leukemic cells. The recent discovery of transition zone/type H vessels that are located close to the endosteal surface and surrounded by osteoprogenitors have shed new light on the composition of this BM region [101]. Further research to elucidate the particular role that transition zone vessels and type H endothelial cells may play in the B-ALL endosteal niche is warranted. The role of endothelial cells in the modulation of B-ALL is discussed further below.

### 3.2. The Central Niche in B-ALL

It is well established that cells constituting the central BM not only create an essential niche for normal B lymphopoiesis, but are also important for the growth, survival and chemoprotection of malignant B cells. Evidence regarding the importance of MSPCs in supporting the progression of B-cell malignancies has come from studies showing that MSCs [102] and BM stromal cells [103] are capable of preventing apoptosis and improving the long-term proliferation of primary B-ALL cells in culture. Indeed, the survival ability of patient-derived primary B-ALL cells on BM-derived stromal cells in vitro has been shown to be a reliable predictor of disease aggressiveness and a patient’s clinical outcome [104]. This indicates that the crosstalk between B-ALL cells and BM stromal cells, such as MSPCs, is an important factor in disease progression and treatment response. Here, we discuss the signaling pathways and the soluble factors in the central niche of B-ALL (Figure 2).

#### 3.2.1. CXCR4/CXCL12 and VLA-4/5-Mediated Mechanisms and Signaling Pathways

The initial homing of B-ALL cells to the central niche is known to be mediated by interactions between CXC chemokine receptor 4 (CXCR4)-expressing B-ALL cells and CXCL12-expressing MSPCs, thus implicating the critical role of the CXCR4/CXCL12 pathway in leukemogenesis. For instance, B-ALL cells are capable of dislodging HSPCs from the niche and disrupting normal hematopoiesis by mediating downregulation of niche-derived CXCL12 [94,105,106], while simultaneously increasing granulocyte colony-stimulating factor expression in MSCs [105,106]. The indispensable role of CXCR4/CXCL12 in B-ALL development is further evidenced by a study that found that expression of phosphorylated CXCR4 in the BM correlated with poorer treatment response and shorter overall survival in adults with B-ALL [107]. In fact, the CXCR4/CXCL12 axis also promotes the survival [108], proliferation [109,110] and dissemination of leukemic cells to peripheral sites around the body [111]. CXCL12 has been found to mediate protection of B-ALL cells from chemotherapeutic agents, with B-ALL cells upregulating CXCR4 expression in response to chemotherapy [112]. Thus, the multifaceted role of the CXCR4/CXCL12 axis in B-ALL renders this pathway an attractive therapeutic target. 

Adhesion of B-ALL cells to MSPCs is further reinforced by the upregulation of cell surface adhesion molecules. For example, a study has observed a reciprocal increase in VLA-4 on the surface of ALL cells, and vascular cell adhesion molecule-1 (VCAM-1), intercellular adhesion molecule-1 and VLA-5 on MSCs following coculture, which also coincided with increasing cell–cell adherence between leukemic cells and MSCs over time [113]. Crucially, high *VLA-4* expression in samples from patients with relapsed B-ALL has been associated with poor overall and event-free survival, thus making VLA-4 a leading target for novel leukemia therapy [114]. Indeed, VLA-4-targeted antibodies such as natalizumab have been shown to significantly impair stromal adhesion in primary B-ALL cells, sensitizing them to chemotherapy and significantly extending the survival of B-ALL-bearing mice [115]. Similarly, it has been reported that disruption of VLA-5 function in BCR-ABL1^+^ leukemic cells using anti-VLA-5 inhibitory antibodies could significantly delay B-ALL engraftment in a xenograft mouse model and act synergistically with imatinib to induce malignant cell apoptosis in vitro [116]. Despite promising preclinical data, little clinical success has been gained with VLA-5 targeted therapies thus far. Various VLA-5 blocking antibodies and small peptides have been developed and tested in multiple cancer subtypes, but have failed to progress beyond phase 3 clinical trials [117]. However, few studies have tested the efficacy of VLA-5 as a therapeutic target in hematological malignancies, which may therefore warrant further investigation.

#### 3.2.2. MSPC-Derived Secretory Factors

Many studies provide compelling evidence that B-ALL cells are able to manufacture and exploit alternative homing pathways to remain in close proximity to MSPCs. This is evidenced in a mouse model of BCR-ABL1^+^ B-ALL, where leukemic cells have been shown to reduce secretion of normal B-cell niche factors (e.g., IL-7 and CXCL12) by mesenchymal progenitors, thus contributing to the disruption of hematopoiesis and the favoring of leukemogenesis [118]. For example, B-ALL cells can induce MSCs to upregulate ActivinA, a transforming growth factor-β family cytokine and a leukemia-promoting factor that mediates both spontaneous and CXCL12-directed migration of B-ALL cells, even in microenvironments with low CXCL12 concentrations [119]. 

The crosstalk between B-ALL cells and BM stromal cells forms the fundamental provision of chemoprotection to B-ALL cells. For instance, BM MSCs harvested from patients with B-ALL have been found to secrete vascular endothelial growth factor (VEGF), driven by the overexpression of heme oxygenase-1 [120]. The same study revealed that VEGF could directly arrest B-ALL cells in the G0/G1 phase of the cell cycle, thus protecting them from cytotoxic drugs that target proliferating cells. Furthermore, BM MSCs from patients with B-ALL exhibit elevated levels of bone morphogenic protein 4 (BMP4) [121]. Of note, this protein has been implicated in the maintenance of acute myeloid leukemia (AML) stem cells and chemoresistance [122]. However, it is currently unclear whether BMP4 plays a similar role in supporting B-ALL cell biology, thus requiring further experimental evaluation. BM stromal cells have also been implicated in mediating ALL cell resistance to cytarabine in vitro via activation of the canonical Wnt signaling pathway within ALL cells [123]. The canonical Wnt signaling pathway is mediated by the accumulation of β-catenin within the cytoplasm, followed by translocation of this protein into the nucleus, where it modulates gene transcription. The addition of a β-catenin inhibitor in combination with cytarabine significantly impaired MSC-mediated cytarabine resistance in ALL cells and significantly increased survival in a xenograft ALL mouse model. This study highlights β-catenin and the Wnt signaling pathway as potential targets for overcoming MSC-mediated chemoresistance [123]. Additionally, galectin-3 has been implicated as a potential chemoprotective factor in B-ALL through its stabilization of β-catenin and activation of Wnt signaling in blast cells [124]. In the BM of patients with B-ALL, expression of galectin-3 was found to be elevated, particularly in relapsed or refractory disease [124]. Furthermore, the induction of galectin-3 upregulation in B-ALL cells in vitro was dependent on MSCs being present in culture, indicating that this protective mechanism is mediated by the BMM [124]. Increased expression of galectin-3 by B-ALL cells has been shown to mediate resistance to both tyrosine kinase inhibitors and vincristine, indicating that therapeutic targeting of this lectin-mediated intercellular communication, in conjunction with standard therapy, could potentially confer advantageous treatment outcomes [125]. 

Insulin-like growth factor binding protein 7 (IGFBP7) has been proposed as a key mediator of treatment resistance to L-asparaginase. In B-ALL, resistance to L-asparaginase is known to be mediated by BM MSCs via upregulation of asparaginase synthetase [126]. In the presence of BM stromal cells, B-ALL cells upregulate IGFBP7 to promote the growth of both B-ALL and stromal cells, as well as to induce the expression of asparaginase synthetase in stromal cells to mitigate L-asparaginase cytotoxicity [127]. Clinical relevance of IGFBP7 expression has also been confirmed by its identification as a negative prognostic indicator associated with poorer leukemia-free survival rates in patients with non-BCR-ABL1^+^ B-ALL [127]. 

Taken together, discovery of these chemoprotective mechanisms is imperative for a better understanding as to why current treatment options do not always lead to an improvement in survival outcomes, particularly for certain subtypes of B-ALL. It is plausible that preventing niche protection of leukemic cells by targeting the secretory pathways of B-ALL-associated MSCs could provide a novel avenue for therapeutic intervention.

#### 3.2.3. Stromal-Derived Extracellular Matrix Proteins

Numerous studies clearly indicate that the BMM is functionally altered by B-ALL cells and exhibits abnormal secretion of a myriad of ECM proteins. One such protein is periostin, which was observed at significantly higher levels in the BM of patients with B-ALL [128]. Periostin was originally identified as an osteoblast-derived adhesion molecule, but is now known to be secreted by a number of BM cell populations to facilitate ECM organization in normal BM [129]. However, over the last decade, deregulated expression of periostin has been recognized as a common feature of many cancers and is believed to contribute to the tumor supportive niche [129]. Notably, a study has demonstrated that B-ALL cells were capable of upregulating periostin expression in BM MSCs, which in turn promoted B-ALL proliferation, adhesion and CC chemokine ligand 2 (CCL2) expression [130]. B-ALL-derived CCL2 could further reinforce periostin expression in MSCs cells, thus establishing a self-reinforcing loop [130]. Periostin disruption in BM MSCs could significantly impair B-ALL development in vivo, demonstrating the potential of periostin as a therapeutic target [130]. 

Matrix metalloproteinase-9 (MMP-9), another key ECM protein in the BM, has also been implicated in B-ALL regulation. BCR-ABL1^+^ B-ALL cells have been reported to induce MMP-9 upregulation in MSCs, resulting in ECM degradation and dissemination of leukemic cells [131]. MMP-9 inhibition in combination with chemotherapy was an effective strategy to increase survival and reduce MRD in a murine model of B-ALL [131]. Lastly, B-ALL cells have been reported to induce MSC upregulation of OPN, which facilitated B-ALL adhesion [94]. Interestingly, the OPN-mediated adhesion mechanism appears to be B-ALL subtype-specific; while the SUP B-15 B-ALL cell line upregulated OPN following interaction with MSCs, REH B-ALL cells did not [94]. Of note, SUP and REH B-ALL cell lines possess different disease-initiating genetic aberrations (expressing *BCR-ABL1* and *ETV6-RUNX1*, respectively), indicating that genetic features may influence the disease microenvironment and thus, the efficacy of microenvironment-targeted therapies. Follow up investigation of OPN in vivo will be worthwhile to elucidate the potential of this protein as a therapeutic target. 

In summary, despite promising preclinical data, there is a lack of clinically-approved, ECM protein-targeted treatments for B-ALL and cancers in general. However, a number of clinical trials evaluating the efficacy of various ECM targeted therapies in solid tumors are currently underway [132]. Future work examining whether any of these targets are applicable to B-ALL will be worthwhile. 

#### 3.2.4. Pro-Inflammatory Cytokines 

Another prominent feature of the malignant central niche is its deregulated inflammatory state. Clinically, serum and plasma samples from patients with B-ALL exhibit increased levels of pro-inflammatory cytokines, including tumor necrosis factor-α (TNF-α), interleukin-6 (IL-6), interleukin-8 (IL-8), interleukin-10, interleukin-12, interferon-γ (IFN-γ) and CCL2 [133,134]. This was further confirmed at a cellular level, where pro-inflammatory cytokines were found to be upregulated in both B-ALL cells [135] and B-ALL-associated MSCs [106,134]. Specifically, pro-inflammatory cytokines elevated in BM MSCs from children with B-ALL included IL-1α, IL-6, interleukin-12p70 and TNF-α [106]. Additionally, coculture of patient derived B-ALL cells and BM MSCs induced IL-8 and CCL2 upregulation in MSCs [134].These cytokines play an instrumental role in maintaining an inflammatory microenvironment that favors leukemogenesis and supports B-ALL malignancy in the central BM niche. For example, a study has demonstrated that CCL2 and IL-8 can increase B-ALL adhesion to MSCs and improve MSC survival in vitro [134]. In addition, a recent study has provided evidence of IL-6 as a therapeutic vulnerability for B-ALL characterized by the *PAX5* mutation [136]. In a native, non-transplant *Pax5* mutant mouse model where B-ALL arises naturally, inhibition of IL-6 with a neutralizing antibody was shown to significantly reduce disease progression [136]. Pro-inflammatory cytokines such as IFN-γ, TNF, IL-1α and IL-1β are also known to enhance MSC-mediated immunomodulation in the BMM [137]. In AML, MSCs in the BMM are known to induce immunosuppression by arresting T cells in G0/G1 phases of the cell cycle, altering T-cell cytokine secretion and enhancing the immunosuppressive capability of regulatory T cells [138]. However, the role of inflammatory-mediated T-cell modulation in B-ALL has not been thoroughly elucidated. A review of the current literature identified a theme of increased regulatory T-cell numbers in patients with B-ALL that, in some cases, possess enhanced immunosuppressive capabilities [139]. While further research is needed, this may indicate that immunosuppression in the central niche may be aiding B-ALL cells to evade detection by the body’s immune system. 

#### 3.2.5. Hypoxia and Hypoxia-Related Mechanisms 

Hypoxia is a prominent characteristic in some areas of the central BM and is known to confer a survival advantage to leukemic cells. While the endosteum was once thought to be the most hypoxic region of the BM, accumulating evidence suggests that in fact, the deep perisinusoidal vascular regions contain the lowest oxygen concentrations [140]. Intriguingly, vast expansion of hypoxic BM regions has been observed with B-ALL progression, and hypoxia-inducible factor 1-α (HIF-1α) was upregulated in both B-ALL and stromal cells extracted from BM biopsies of patients with B-ALL [141]. Interactions between MSCs and B-ALL were shown to be capable of promoting HIF-1α activation in B-ALL cells [142]. This resulted in activation of the AKT/mTOR pathway and a metabolic switch to glycolysis, conferring chemoprotection to leukemic cells [142]. Thus, inhibition of HIF-1α may present a viable strategy to perturb disease progression and induce leukemia chemosensitivity. Alternatively, others have proposed that harnessing the hypoxic quality of the malignant central niche by using hypoxia-activated cytotoxic drugs may allow more targeted delivery of therapeutics to leukemic cells [141].

#### 3.2.6. Tunneling Nanotubes and Extracellular Vesicles

Recent advances in leukemia research have discovered new and exciting crosstalk mechanisms that are utilized by B-ALL cells to alter BM niche cells and establish a disease permissive microenvironment. Polak et al. were the first to identify the presence of tunneling nanotubes, mediating intercellular communication between B-ALL cells and BM MSCs [143]. This cellular interaction induced MSC secretion of pro-inflammatory cytokines and imparted chemoprotection to B-ALL cells in the presence of prednisolone [143]. Subsequent studies have further delineated these tunneling nanotubes to be instrumental in the intercellular transfer of mitochondria, autophagosomes and adhesion molecules, as well as driving secretion of pro-survival cytokines from MSCs [144]. In addition, primary B-ALL cells and B-ALL cell lines have been observed to release extracellular vesicles that are internalized by BM stromal cells [145]. The nature of these extracellular vesicles is still largely unknown, but they appear to be anucleate and contain intact organelles (e.g., mitochondria and lysosomes) and an organized cytoskeleton [145]. Internalization of these vesicles appears to provoke a glycolytic metabolic shift in stromal cells, resulting in the induction of extracellular lactate release, which may support leukemic cell survival and chemoresistance under oxidative or cytotoxic stress [146]. Collectively, these new intercellular communication mechanisms are worthy of investigation for their potential as novel therapeutic targets in B-ALL. 

#### 3.2.7. Endothelial Cells 

Angiogenesis has long been known to promote the growth and survival of solid tumors; however, its significance in the progression of hematological malignancies has become increasingly appreciated in recent years [147]. The importance of the vascular endothelium in B-ALL begins at disease engraftment, when B-ALL cells migrate to the endothelium via E-selectin and CXCL12 interactions [148,149]. Following adhesion of B-ALL cells to endothelial cells in vitro, endothelial cells were found to upregulate their expression of VCAM-1 and engage with B-ALL cells through activation of VCAM-1 signaling pathways [94]. Further in vitro data indicates that these endothelial interactions promote B-ALL cell survival by stimulating expression of the antiapoptotic factor BCL-2 [150]. 

Following localization within the perivascular niche, leukemic cells can further remodel the vasculature into a leukemia-supportive network to promote leukemogenesis in the BM. In fact, increased microvessel density and complexity has been observed in the BM of patients with B-ALL compared to healthy controls [151,152]. In corroboration with these findings, a study has detected an increase in CD31^+^ endothelial cell frequency in the BM of a mouse model of B-ALL [153]. Notably, pro-angiogenic factors including basic fibroblast growth factor [150,151,154,155], hepatocyte growth factor and TNF-α [155] are heightened in urine and plasma samples of patients with ALL. Furthermore, addition of plasma from patients with ALL to endothelial cells in Matrigel stimulated proliferation, migration and capillary-like structure formation, thus confirming the functional capability of proangiogenic factors in the B-ALL BM [150]. 

A major function of the BM vasculature is trans-endothelial migration (TEM), which controls the transit of cells (e.g., immature and mature lymphocytes) from the BMM to the periphery and vice versa through the sinusoids [156]. TEM is also an important mechanism for dissemination and infiltration of B-ALL cells to peripheral organs in later stages of disease. Factors essential for TEM include cortactin [157], the formin mDia1 [158] and VEGF [159]. Upregulation of cortactin has been observed in B-ALL cells, with greater expression correlated with disease infiltration into peripheral organs in PDX models [157]. Additionally, samples from patients with relapsed B-ALL exhibited threefold greater expression of cortactin than newly diagnosed patients, highlighting the clinical relevance of this factor [157]. VEGF upregulation was also associated with central nervous system infiltration in PDX models of ALL [159]. This has led to the hypothesis that pharmacologically reducing the levels of these factors may be an effective method to reduce the invasiveness and spread of B-ALL. Indeed, knock-down of cortactin in B-ALL cells has been shown to inhibit disease establishment and infiltration of blast cells in peripheral organs in mice [157]. Similarly, knock-down of mDia1 in B-ALL cells significantly reduced leukemia progression in vivo and prolonged survival [158]. While these preclinical results appear promising, the efficacy of pharmacologically manipulating TEM factors is yet to be clinically tested. 

#### 3.2.8. Adipocytes

In recent years, the role of adipocytes in the pathogenesis of acute leukemias has been characterized [160,161]. In particular, adipocytes have been implicated in the regulation of B-ALL. For example, an in vivo study observed CXCL12-mediated homing of B-ALL cells to visceral fat from early stages of disease, which was significantly amplified in obese mice [162]. In line with these findings, it has been reported that extramedullary BM, derived from CTGF-deficient MSCs that exhibit a differentiation bias towards the adipogenic lineage, could generate an adipocyte-rich niche that facilitates B-ALL cell homing and engraftment [163]. This adipocyte-driven leukemic cell homing was also postulated to be driven by CXCL12 and leptin in the niche environment [163]. Of note, upregulation of CTGF has actually been observed in the BM and peripheral blood of patients with B-ALL [164] and CTGF is highly expressed by B-ALL cells [165]. Thus, while CTGF deficiency in MSCs was useful for generating an adipo-lineage bias in this model, a reduction in CTGF levels does not recapitulate the B-ALL BMM. Nevertheless, these results signify that an important regulatory communication exists between adipocytes and B-ALL during leukemogenesis; however, the precise mechanisms have yet to be clearly defined. Interestingly, one of the purported mechanisms with which adipocytes support B-ALL cells is through provision of free fatty acid release via lipolysis [166]. However, others have suggested that human BM adipocytes possess altered lipolytic activity, thus raising the possibility that in humans, other non-free fatty acid mechanisms between BM adipocytes and B-ALL may be at play [167]. 

Another key mechanism by which adipocytes can significantly modulate B-ALL cells in the BMM is via release of secretory factors. Adipocytes secrete a myriad of adipokines, such as IL-6, IGF-1, TNF-α, adiponectin, leptin and resistin [168]. Leptin and resistin levels are elevated in children with B-ALL at diagnosis, while adiponectin levels are found to be reduced [169]. Leptin is known to promote the growth of myeloid leukemic cells [170], but its role in B-ALL is less clear. A recent report shed light on the role of leptin and LepR signaling in adipocyte-B-ALL crosstalk, where adipocyte-rich niches, which are abundant in leptin, could attenuate the expression of LepR on the surface of B-ALL cells [171]. This was hypothesized to lead to a reduction in LepR-activated signaling cascades that would normally drive the terminal differentiation of B-cell progenitors, and instead favor the maintenance of the malignant blast population. While strong LepR expression on leukemic cells is a good prognostic indicator in patients with B-ALL, it is important to note that B-ALL cells generally express LepR at lower levels than normal lymphocytes [171]. Thus far, the clinical use of LepR or its signaling-related genes as reliable prognostic indicators remains to be evaluated. 

Intermittent fasting, which is a dietary-based treatment approach that has garnered some preclinical success in the solid tumor field [172], was recently demonstrated to be a novel treatment method capable of disrupting leptin-mediated support of B-ALL in mice [171]. Analysis of a fasting regimen, which consisted of alternate feeding and fasting days in a mouse model of B-ALL, exhibited striking inhibition of leukemia development and even induced reversal of mid-late-stage disease [171]. While fasting has been reported to increase marrow adipose tissue [173], this method also contributes to reduced leptin levels in the BM [171]. Mechanistically, fasting-induced depletion of leptin is hypothesized to cause a compensatory increase in B-ALL LepR surface expression and activation of LepR-signaling pathways, leading to increased terminal differentiation of B-cell progenitors and subsequently diminishing the malignant blast population [171]. This treatment, termed ‘differentiation therapy’, could potentially be used as a novel treatment approach for B-ALL.

Interestingly, a comprehensive set of functional studies have also highlighted the powerful chemoprotective role of adipocytes in B-ALL. In children with B-ALL, obesity is known to be associated with an increased risk of persistent MRD and an inferior event-free survival [174]. In support of this, obesity in murine models was found to significantly decrease the efficacy of chemotherapeutic agents, with B-ALL cells observed to take refuge in adipose tissue both in vitro and in vivo [162,175,176]. Several studies have delineated the chemoprotective mechanisms conferred by adipocytes on B-ALL cells. For instance, adipocytes have been shown to prevent chemotherapy-induced apoptosis in B-ALL cells by upregulating anti-apoptotic factors, BCL-2 and PIM-2 [175]. In addition, accumulating evidence suggests that B-ALL cells can induce an adipocyte oxidative stress response, which facilitates the protection of B-ALL cells from daunorubicin-induced cytotoxicity through secretion of protective factors [177]. Further investigation also uncovered the capability of adipose tissue to absorb and metabolize daunorubicin, effectively decreasing cytotoxicity in the leukemia microenvironment [178]. Finally, expansion of the adipocyte population and upregulation of glutamine synthetase in adipocytes was noted in patients with high-risk ALL following induction chemotherapy [176]. Increased production of glutamine synthetase is suspected to reduce the efficacy of L-asparaginase, which is a chemotherapeutic agent that depletes glutamine and asparaginase, amino acids that are essential for leukemic cell survival, from the leukemia niche. In vitro investigation found that dexamethasone, a glucocorticoid given during induction chemotherapy, was able to induce glutamine synthetase upregulation in adipocytes, which may explain findings in humans [176]. The higher frequency of adipose tissue resulting from obesity may therefore magnify this chemoprotective effect. In support of this theory, obese mice responded worse to treatment with L-asparaginase following implantation with B-ALL cells, compared to lean mice [176]. 

Collectively, these studies suggest that B-ALL cell migration to adipocyte-rich niches imparts chemoprotection and therefore may contribute to MRD and relapse. Mitigating these disease supportive mechanisms and the expansion of the BM adipocyte population following current treatment regimens may improve outcomes for patients with this disease. 

## 4. Emerging Techniques and Technologies

While the field of BMM research has made substantial progress in recent decades, developing novel and innovative experimental techniques capable of capturing the tremendous complexity in this biological system is critical to our understanding of leukemia progression. For instance, previously developed 2D and 3D in vitro models have failed to comprehensively recapitulate the vast complexity of interactions occurring in BM niches. However, the recent development of a microfluidic system termed “leukemia-on-a-chip” has been described as a “game changer” solution to this problem. The sequential loading of BM cells into different compartments in this system recapitulates the spatial relationship between cells in the BMM. Furthermore, this model is capable of mimicking normal and leukemic BMMs by creating a dynamic environment that contains concentration gradients, fluid sheer stress and mechanical stress [94]. This biomimetic system has many advantages and could provide a high-throughput platform for the investigation of BM and leukemic cell interactions and niche-targeted therapies prior to testing in vivo. As highlighted by the authors, this has the potential to reduce inter-sample variability in biological parameters that cannot be controlled when using animal models, and allow easy, real-time visualization of spatiotemporal relationships between BMM components via microscopy. Additionally, this methodology could potentially be used for the application of personalized medicine, allowing optimization of selective niche-targeted therapies using cells derived from patients.

Over the years, emerging imaging [179] and sequencing technologies [180] have shown immense promise in being able to capture processes occurring in the B-ALL BMM at a finer spatial and temporal resolution, allowing substantial improvement in our understanding of B-ALL niche biology. For example, intravital microscopy allows real-time analysis of the BMM with the ability to capture cellular processes such as cellular engraftment, adhesion, migration and apoptosis in vivo. Additionally, more recently developed techniques such as imaging mass cytometry will further increase the detail that can be resolved, with vast panels of markers capable of being detected in a single tissue section [181]. The increasing availability and feasibility of single cell RNA sequencing [182] and spatial transcriptomics will also enable a better understanding of changes occurring at the molecular level of individual BM niche populations at multiple stages of disease. 

It is also important to acknowledge that precise elucidation of B-cell niche populations has been hampered by the lack of specificity in promoters used to drive recombination in transgenic mouse models, where current reporter and conditional knockout strains target a heterogeneous population of BM niche cells. For example, the *Ocn-Cre*, *Dmp1-Cre* [183] and *Osx-Cre* [22] models target CAR cells in addition to targeting osteolineage cells. In fact, 70% of CAR cells and arteriolar pericytes are targeted by *Ocn-Cre* transgenes [183]. Current models targeting osteolineage populations also fail to recapitulate their complexity. For example, single cell RNA sequencing has identified that the COL2.3^+^ osteoblast population actually consists of three transcriptionally distinct populations [29]. Therefore, the development of models with enhanced genetic manipulation capability that can specifically target more homogenous niche populations is necessary. In the central niche, many common MSPC markers are also detected in differentiated stromal populations. For example, the *Prx1-Cre* transgene has also been found to target osteolineage cells including osteocytes and mature osteoblasts [22]. Similarly, the *Tie2-Cre* model, while used to target endothelial cell populations, has been observed to also target hematopoietic populations [184]. Thus, these findings caution against the classification of results obtained using these models as strictly confined to a single endosteal or central niche cell population. Ultimately, enhancing the specificity of current gene manipulation in mouse models will be important for future research. For instance, use of the relatively new CRISPR/Cas9 system to engineer new transgenic mouse models may provide one solution to this problem, as it allows precise targeted modifications to the genome, thus improving the efficiency and simplicity with which transgenic models can be engineered [185]. 

## 5. Conclusions

Recent decades have seen a vast expansion in our understanding of the role that BM niche cells play in hematopoiesis and hematological malignancies. At the expense of B lymphopoiesis, B-ALL cells exploit niche-supportive signaling and remodel the normal B-cell niche to create a microenvironment that is supportive of leukemic cell expansion, while also providing chemoprotective niches that foster MRD and subsequently disease relapse. Advances in standard therapies used to treat B-ALL have seen five-year survival rates improve to over 90%. However, patients with high-risk genetic subtypes, such as the *BCR-ABL1* translocation and *KMT2A*-rearrangements, and relapsed B-ALL have a significantly inferior response to standard treatments [186,187]. In addition, increasing the dose intensity of chemotherapy regimens does not necessarily impart a survival benefit to these patients due to toxicity-associated deaths [188]. Previously, many leukemia therapies were developed based on targeting leukemic cell-intrinsic attributes at the molecular level, such as cell cycle regulatory proteins, constitutively activated tyrosine kinase or cytokine receptor signaling, as well as genetic alterations that drive cell malignancy. In recent years, accumulating evidence increasingly supports the concept of therapeutically targeting the cell-extrinsic interactions between leukemic cells and BM niche cells within the BMM to enhance conventional chemotherapy [189]. Agents which target leukemic cell engraftment and chemoresistance such as CXCR4/CXCL12 inhibitors, Wnt signaling inhibitors, Notch inhibitors, as well as VLA-4 and E-selectin antagonists, have shown promising therapeutic efficacy either in preclinical studies and/or clinical trials [189]. Despite this, there is a lack of new, BMM-targeted therapeutic agents either being approved by the FDA or currently undergoing clinical trials for the treatment of B-ALL. Therefore, devising and testing therapeutics that target the BMM of B-ALL will be imperative for improving clinical outcome for patients, particularly in those with poor prognoses.
